# Neutrophil Extracellular Traps Are Found in Bronchoalveolar Lavage Fluids of Horses With Severe Asthma and Correlate With Asthma Severity

**DOI:** 10.3389/fimmu.2022.921077

**Published:** 2022-07-13

**Authors:** Pierre Janssen, Irene Tosi, Alexandre Hego, Pauline Maréchal, Thomas Marichal, Coraline Radermecker

**Affiliations:** ^1^ Laboratory of Immunophysiology, GIGA Institute, Liège University, Liège, Belgium; ^2^ Faculty of Veterinary Medicine, Liège University, Liège, Belgium; ^3^ In Vitro Imaging Platform, GIGA Institute, Liège University, Liège, Belgium

**Keywords:** neutrophil extracellular traps, severe asthma, moderate asthma, horses (Equus Caballus), neutrophil, equine asthma physiopathology, equine asthma biomarker

## Abstract

Asthma encompasses a spectrum of heterogenous immune-mediated respiratory disorders sharing a similar clinical pattern characterized by cough, wheeze and exercise intolerance. In horses, equine asthma can be subdivided into severe or moderate asthma according to clinical symptoms and the extent of airway neutrophilic inflammation. While severe asthmatic horses are characterized by an elevated neutrophilic inflammation of the lower airways, cough, dyspnea at rest and high mucus secretion, horses with moderate asthma show a milder neutrophilic inflammation, exhibit intolerance to exercise but no labored breathing at rest. Yet, the physiopathology of different phenotypes of equine asthma remains poorly understood and there is a need to elucidate the underlying mechanisms tailoring those phenotypes in order to improve clinical management and elaborate novel therapeutic strategies. In this study, we sought to quantify the presence of neutrophil extracellular traps (NETs) in bronchoalveolar lavage fluids (BALF) of moderate or severe asthmatic horses and healthy controls, and assessed whether NETs correlated with disease severity. To this end, we evaluated the amounts of NETs by measuring cell-free DNA and MPO-DNA complexes in BALF supernatants or by quantifying NETs release by BALF cells by confocal microscopy. We were able to unequivocally identify elevated NETs levels in BALF of severe asthmatic horses as compared to healthy controls or moderate asthmatic horses. Moreover, we provided evidence that BALF NETs release was a specific feature seen in severe equine asthma, as opposed to moderate asthma, and correlated with disease severity. Finally, we showed that NETs could act as a predictive factor for severe equine asthma. Our study thus uniquely identifies NETs in BALF of severe asthmatic horses using three distinct methods and supports the idea that moderate and severe equine asthma do not rely on strictly similar pathophysiological mechanisms. Our data also suggest that NETs represent a relevant biomarker, a putative driver and a potential therapeutic target in severe asthma disease.

## Introduction

The term “asthma” is widely used to refer to a state of immune-mediated chronic inflammation of lower respiratory airways sharing a common clinical presentation characterized by chronic cough, excessive mucus secretion, bronchial hyperresponsiveness and exercise intolerance with variable degrees of severity (1, 2). In recent years, human physicians and researchers have invested efforts to determine asthma phenotypes and endotypes based on various criteria such as the cellular composition of sputum, the underlying immune response, triggering factors or response to therapy (3). In horses, two asthma phenotypes have been described according to clinical presentation and history, endoscopy findings and BALF cytology (4, 5). Severe asthma, formerly known as recurrent airway obstruction (RAO), affects around 14% of adult horses (>7 years) (6) that present chronic cough, exercise intolerance, labored breathing at rest, high mucus score at endoscopy (>3/5) and high neutrophil counts in BALF (4, 5, 7). Mild or moderate asthma, formerly known as inflammatory airway disease (IAD), is frequently diagnosed in horses of all ages that show subtle symptoms such as poor performance, occasional coughing but normal breathing at rest. Of note, such asthma phenotype has been reported in 68-77% of thoroughbred horses (4, 8, 9). This form can be associated with intermediate mucus secretion and divided into distinct sub-phenotypes according to the type of infiltrating inflammatory cells in the airways (10–12). Indeed, moderate asthmatic horses can show mild increased neutrophil influx or mild increased mastocytic or eosinophilic influx in the lower airways requiring sensitive detection methods (9–11). Investigating the pathophysiology of these two forms of the disease and their sub-classification would allow the elaboration of more targeted therapies and an improved clinical management of affected horses for practitioners. Yet, the immune mechanisms governing the pathophysiology of distinct disease phenotypes are still poorly understood and represent an unmet medical need.

Neutrophils are innate immune cells easily recognizable thanks to their plurilobular nucleus and numerous cytoplasmic granules storing cytotoxic compounds (13). Neutrophils are one of the first cells recruited upon pathogen invasion in tissues. Indeed, they can counteract pathogen expansion by their potent phagocytic activity, the exocytosis of their cytotoxic granules or the release of neutrophil extracellular traps (NETs) (14). The latter neutrophil function has been originally described by Zychlinsky and his team in 2004 (15). NETs are extracellular structures composed of nuclear or mitochondrial DNA associated to citrullinated histone 3 (Cit-H3) and decorated with various antimicrobial peptides such as myeloperoxidase (MPO), neutrophil elastase (NE), LL-37, cathelicidins, etc (14). NETs can be released *in vivo* upon stimulation by a wide range of stimuli like bacteria (15), viruses (16–18), parasites (19, 20), immune complexes (21) or crystals (22). They can catch pathogens to avoid their systemic spreading and also directly destroy them thanks to their antimicrobial properties (14). Aside this beneficial role, excessive NETs production or defective NETs elimination have also been described to have harmful effect in immune-mediated disorders such as rheumatoid arthritis (23), anti-neutrophil cytoplasmic autoantibody-associated vasculitis (24), atherosclerosis (25) and coagulopathies (26, 27). Moreover, NETs have been identified as potential key players during the onset of environment-driven allergic asthma in mice (28) and in the pathophysiology of human neutrophilic asthma (29) and chronic obstructive pulmonary disease (COPD) (30). Several methods have been developed to detect and quantify NETs in biological fluids, in cells in culture and in tissue section, mostly in human and mice (31, 32) but also in the horse species (33–35).

The immunological mechanisms underlying equine asthma are still largely unknown. Disease development seems to rely on complex interactions between host genetic factors and environmental factors, such as exposure to allergens or other airborne particles (36). In many cases, equine asthma is triggered by exposure to environmental allergens, and asthmatic horses display a predominant neutrophilic inflammation of the lower airways and a mixed immunological profile characterized by both type 2 (i.e., IL-5 (37), IL-4 (37, 38)) and type 17 [IL-17 (39, 40)] cytokines (7, 41). Indeed, neutrophils are recruited to the airways of horses upon allergenic hay challenge and constitute one cardinal feature used to make a diagnosis of equine asthma when they are found in BALF samples of horses with respiratory symptoms or poor performance (42). Severe asthmatic horses usually show high BALF neutrophil counts (>20% of total BALF cells) (12), whereas moderate asthmatic horses tend to display moderately increased neutrophil counts (6-20%) in their BALF (12). Neutrophil levels have also been directly correlated with the severity of pulmonary lesions during asthma exacerbations in horses (43). Moreover, blood neutrophils of asthmatic horses display unique features such as a high respiratory burst activity (44), prolonged *ex vivo* lifespan and lower bactericidal activity (45). Interestingly, blood of severe asthmatic horses contains a higher proportion of low-density neutrophils as compared to healthy controls, which is no longer observed in horses in remission, suggesting a correlation between low-density neutrophils and disease status in severe equine asthma (35). Of note, low-density neutrophils have been described to be particularly prone to release NETs (46–48). NETs have been identified by several studies as important players in respiratory disorders of the lower airways in man (49) and mice (28, 50, 51), where they can induce important damage of the lung tissue (52, 53), aberrantly activate other innate immune cells (25, 54–56) or favor thrombi formation (26). In asthma, NETs can facilitate the induction of type 2 immunity associated with allergic asthma (28, 50) and favor differentiation of helper type 17 T (Th17) cells (57) and skewing toward neutrophilic inflammation in neutrophilic asthma (58). Despite the increasing body of evidence regarding the implication of NETs in the pathophysiology of human asthma, only few studies have investigated the presence of NETs in the airways of severe equine asthma (33–35) and the detection of NETs in the horse species currently lacks standardization. Finally, while NETs have already been observed in severe equine asthma (33–35), their presence in moderate equine asthma has not been investigated yet.

In this study, we hypothesized that NETs, when adequately quantified using standardized methods in BALF samples, could be differentially detected in both equine asthma phenotypes and could be a biomarker of one asthma phenotype, which would suggest distinct underlying pathophysiological mechanisms. First, we optimized NETs detection in horses using *ex vivo*-stimulated blood neutrophils by measuring cell-free (cf) extracellular DNA and MPO-DNA complexes in culture supernatants, or by directly visualizing and quantifying NETs areas by confocal microscopy. Next, using these methods, we compared NETs release in BALF samples of healthy controls, moderate or severe asthmatic horses and identified NETs as a specific feature of severe asthma that correlated with disease severity and could act as a predictive biomarker for the severe asthma phenotype.

## Material and Methods

### Animals

Owners gave informed consent for their animals’ inclusion in the study. Horses with equine asthma were enrolled from animals referred to the Faculty of Veterinary Medicine of the University of Liège for poor performance, or specific respiratory symptoms, between December 2020 and March 2022. Of note, asthmatic horses recruited in this study were diagnosed with “hay dust asthma” solely, as they were mainly sport horses, showing symptoms when stabled indoor and with generally no access to pasture or very limited paddock turnout. Horses were included in the equine asthma group based on the evidence of respiratory disease at the history and/or at the physical exam and on the increase of at least one granulocytic population (neutrophils >5%, eosinophils >1%, mast cells >2%) upon evaluation of BALF cytology. Exclusion criteria for asthmatic horses were an increased rectal temperature (>38.5°C), a positive bacterial culture on the tracheal wash (TW) a recent (within 10 days before presentation) treatment with corticosteroids and/or bronchodilators. Distinction between severe equine asthma and moderate equine asthma was made using several criteria: breathing pattern at rest, lung auscultation and BALF cytology. The observation of a labored breathing, an abnormal lung auscultation or a BALF neutrophilia >20% were considered indicative of severe equine asthma. Absence of a modified lung auscultation and of an increased breathing effort, together with a moderate increase in neutrophil percentage (5-20%) and/or in eosinophils and/or in mast cells were considered consistent with moderate equine asthma. The sole cutoff of BALF neutrophils was not considered as a sufficient criterion for the distinction between severe and moderate equine asthma, because a poor correlation has been described between diagnostic results (i.e., based on BALF cytology) and clinical signs in horses with equine asthma (7). Control horses were recruited from the teaching horse herd of the University of Liège, from horses owned by staff members of the University of Liège and from horses referred to the University for routine check-up. Inclusion criteria for control horses were the absence of any history or clinical finding indicating potential respiratory problems (e.g., cough, nasal discharge, fever, poor performance, increased respiratory effort, abnormal respiratory auscultation); a normal physical and cardio-respiratory examination; a BALF cytology within reference values (neutrophils ≤5%, eosinophils ≤1%, mast cells ≤2%) as described elsewhere (59) and a negative bacterial culture on the TW. The characteristics of the subjects included in the study are presented in **Tables 1**, **2** for healthy controls and asthmatic horses, respectively. Weighted clinical score (WCS) data have been attributed to asthmatic subjects based on the WCS criteria published by Lavoie and colleagues (60).

**Table 1:** Cytology findings of healthy horses enrolled in this study.

	BALF cytology (%)				
	Macrophages	Lymphocytes	Neutrophils	Eosinophils	Mast cells
Healthy controls	63	33.5	1.5	0	2
	47	48	4	0	1
	49.5	43.5	5	0	2
	44	53.5	0.5	0	2
	41	54	4.5	0	0.5
	32.5	63	3.5	0	1
	44.5	50.5	3	0	2
	34.5	61.5	3.5	0	0.5
	47	45.5	5.5	0	2
	44	53	1.5	0	1.5
	39.5	50	8	0.5	2
	42.5	53	4	0	0.5

**Table 2:** Clinical characteristics, airway mucus and cytology findings of asthmatic horses enrolled in this study.

Group	Complaint	Abnormalities at the physical exam	Labored breathing at rest	Tracheal mucus score (/V)	Tracheal neutrophilia (>20%)	WCS (/23)	BALF cytology (%)				
							Macrophages	Lymphocytes	Neutrophils	Eosinophils	Mast cells
SEA	Cough and poor performance	Nasal discharge	yes	IV	yes	10	42	33	24	0	1
	Cough, nasal discharge, poor performance	Cough reflex, increased lung sounds	no	V	yes	9	27.5	30.5	40	0	2
	Cough, nasal discharge, poor performance	Lung crackles and wheezes	yes	V	yes	23	4	4	92	0	0
	Cough, nasal discharge, poor performance	Lung crackles and wheezes	yes	IV	yes	14	26,5	50,5	14	0	9
	Cough and nasal discharge	Nasal discharge	yes	II	yes	7	54	24	20	0	2
	Cough and nasal discharge	Nasal discharge	no	III	yes	6	34	23	43	0	0
	Cough and nasal discharge	Nasal discharge	yes	III	yes	8	39	25	35	0	1
	Cough, nasal discharge, poor performance	None	yes	IV	yes	5	22	29	49	0	3
	Cough	Cough reflex, increased lung sounds	no	IV	yes	6	23	47.5	28	0	1.5
	Cough and nasal discharge	Nasal discharge, increased lung sounds	no	V	yes	13	17	10	73	0	0
	Cough and nasal discharge	Nasal discharge	yes	IV	yes	6	34.5	22.5	42	0	1
	Cough and nasal discharge	Increased lung sounds	no	V	yes	8	34.5	18	46	0	1.5
	Cough	Cough reflex	yes	III	yes	5	30	44	25	0	1
	Cough and poor performance	Cough reflex	no	III	NA	4	19	57	24	0	0
MEA	Poor performance	None	no	II	no	0	33.5	60.5	1.5	0	4.5
	Poor performance	None	no	II	no	0	34.5	48	15	0	2.5
	Cough, poor performance	None	no	II	no	2	68	27	1	0	4
	Cough	Nasal discharge	no	II	no	3	51	28	19	0	2
	Poor performance	None	no	II	yes	0	42.5	40	14	0	3.5
	Poor performance	Cough reflex	no	III	yes	1	45.5	46	8	0.5	0
	Poor performance	Cough reflex and nasal discharge	no	II	no	2	55	39	1	1.5	3.5

SEA, severe equine asthme; MEA, moderate equine asthma.

All the procedures have been approved by the local Ethical Committee (File references DE2453 for BAL and TW procedures and DE1565 for blood collection).

### Reagents and Antibodies

A complete list of the reagents, antibodies and software used in this manuscript can be found in **Table 3**.

**Table 3:** List of antibodies, reagents and software used in this study.

Antibodies	Source	Cat#
4’,6-diamidino-2-phénylindole (DAPI)	ThermoFisher	D3571
Anti-goat IgG (H+L) Polyclonal Antibody (Donkey), AF488 conjugated	ThermoFisher	11055
Anti-mouse DNA Monoclonal Antibody (Rat, clone BV16-13), unconjugated	Sigma	MAB030
Anti-mouse IgG2a Biotin Antibody (Rat)	BD Biosciences	553388
Anti-mouse/human Histone H3 (citrulline R2+R8+R17) Polyclonal Antibody (Rabbit), Unconjugated	Abcam	Ab5103
Anti-mouse/human Myeloperoxidase/MPO Polyclonal Antibody (Goat), Unconjugated	R&D Systems	AF3637
Anti-rabbit IgG (H+L) Polyclonal Antibody (Donkey), AF568 conjugated	ThermoFisher	A10042
Avidin-HRP	ThermoFisher	18-4100-94
**Chemicals, Peptides and Recombinant Proteins**	**Source**	**Cat#**
3,3’,5,5’ Tétraméthylbenzidine (TMB)	Life technologies	SB02
Ammonium chloride (NH_4_Cl)	VWR chemicals	21236267
Bovine Serum Albumin (BSA)	Sigma	A7906
Detomidine (Domidine^®^)	Alcyon	2401677
DMEM, low glucose, pyruvate, no glutamine, no phenol red	Gibco	11580406
DNase I	Sigma	11284932001
dNTP	ThermoFisher	N8080260
DPBS	ThermoFisher	14190094
EDTA	Merck Millipore	1084181000
Fetal Bovine Serum (FBS)	Sigma	F7524
Formaldehyde 37%	Fisher	10532955
Percoll	GE Healthcare	#17089101
Phorbol myristate acetate (PMA)	Sigma	P1585
Poly-D-lysine hydrobromide	Sigma	P6407
Potassium hydrogen carbonate (KHCO_3_)	ThermoFisher	448015000
ProLong™ Diamond Antifade Mountant	ThermoFisher	P36961
Quant-iT™ PicoGreen™ dsDNA Assay Kit	ThermoFisher	P11496
Triton X-100 Detergent	Merck	648466
Tween-20	ThermoFisher	233360010
NH_4_Cl	VWR	12125-02-9
KHCO_3_	Fluka	331051/1
**Software and algorithms**	**Source**	**URL**
Adobe Illustrator 2022	Adobe	
ImageJ	ImageJ Software	https://imagej.net/
Prism 9	GraphPad Software	https://www.graphpad.com/scientific-software/prism/

### Sample Collection and Processing

Horses were sedated with detomidine (10-20 µg/kg IV) for endoscopic evaluation of the respiratory tract. A 2.50 m-long endoscope (Karl Storz, Utrecht, Netherlands) was inserted through the nasal cavity, then passed into the trachea until it was wedged.

*Tracheal wash:* A sterile plastic and plugged catheter was inserted through the biopsy channel of the endoscope approximately 3 cm away from the distal end. Tracheal mucus was assessed and scored using a 0-5 scale, as previously described (61). Then, the endoscope was retracted to the rostral trachea and the plastic catheter was advanced to be readily observed protruding from the distal end of the endoscope. A volume of 20 ml of pre-warmed sterile saline (0.9% NaCl) was instilled onto the tracheal mucosa. The catheter was retracted back into the endoscope and the latter was advanced until a pool of saline mixed with secretions was visualized near the thoracic inlet. The catheter was exposed once more to collect the pool of fluid, then transferred to a sterile container and submitted for cytology and bacteriology performed by a referenced laboratory.

*Bronchoalveolar lavage:* After the endoscope was wedged, BAL was performed by instillation and subsequent re-aspiration of a volume of 250 mL of sterile saline (0.9% NaCl) pre-warmed to room temperature in water bath. We discarded cases with BAL return volumes considered too low, i.e., lower than 50% of the originally instilled volume. When possible, the BAL procedure was performed twice, passing the endoscope through both primary bronchi, thus the two BALFs were pooled together for cytology as suggested in the literature for the diagnosis of equine asthma (62). The BALF samples were mixed on a rotational mixer and they were kept refrigerated until processing. The cytospin preparations were made by adding 150 µL of BALF to a cytospin funnel, which was then centrifuged at 1000 rpm (112 x *g*) for 4 minutes (rotor JC 301 Cellspin I 1206-14, Tharmac). Slides were stained with May-Grünwald-Giemsa (MGG). A differential cell count was performed on 300 cells; each cell type was counted as a percentage of total nucleated cells. The remaining BALF volume of each sample was centrifuged twice for 10 minutes in a countertop centrifuge at 1600 rpm (541 x *g*, rotor S-4-104 Centrifuge 5810R, Eppendorf, at 24°C, brake 5, accel 7). Six aliquots of 1.5 ml of supernatant were retained per sample. The remaining supernatant was discarded and the cell pellets and supernatants were stored at -80°C. All samples were processed within 2 hours after collection.

### Blood Neutrophil Isolation and *Ex Vivo* Stimulation

Venous blood was collected from the jugular vein of 3 healthy horses using a vacutainer^®^ and a needle (0.9 x 38 mm 20G). Blood was collected in 9 ml EDTA tubes. The tubes were centrifuged (1400 rpm or 414 x *g*, 7 min, rotor S-4-104 Centrifuge 5810R, Eppendorf) to isolate the plasma. A volume of 1 ml of blood cells was collected and added to 5 mL of hemolysis solution (8,26 g/l NH_4_Cl, 1g/l KHCO_3_, 37 mg/l EDTA in distilled water, pH 7,4) for a period of 5 min, followed by the adding of 5 ml of PBS-EDTA (10 nM, pH 7,4). This mixture was left at room temperature for 10 min before being centrifuged (1400 rpm or 414 x *g*, 7 min, 21°C, rotor S-4-104 Centrifuge 5810R, Eppendorf). After centrifugation, the pellet was re-suspended in 5 ml of 1,080 density Percoll, then 3 ml of 1,038 density Percoll and 2 ml PBS were added without mixing the distinct fractions. A centrifugation was performed without brake (1600 rpm or 541 x *g* with rotor S-4-104 Centrifuge 5810R, Eppendorf, 20 min, 21°C) to avoid mixing of the different fractions. Neutrophils were present at the interface between 1,080 and 1,038 densities and were collected carefully. Purity was >80% as measured by a differential cell count after Diff-Quick staining. We then determined neutrophil numbers and resuspended them to a concentration of 2.10^6^ cells/ml in DMEM medium (Gibco, 11580406). Neutrophils were seeded in 8-well plates (2,5 ml per well, Sigma, C7057-1PAK) and were left unstimulated (negative control) or stimulated with 0,05 µg/ml phorbol 12-myristate 13-acetate (PMA, Sigma, P1585) for 3 hours at 37°C and 5% CO_2_ (positive control). After incubation, supernatants were collected. Wells were then rinsed twice with 3 ml PBS and collected. Both supernatants and PBS were pooled, and stored at -80°C in 1.5 ml aliquots.

### Quantification of Cell-Free DNA (cf DNA)

To estimate the abundance of cf DNA in the supernatant, we used the Quant-iT PicoGreen dsDNA Assay Kit (Invitrogen, Carlsbad, CA, P7589) according to the manufacturer’s instructions. Briefly, a DNA standard curve (from 0.4 µg/ml to 3.125.10^-3^ µg/ml) was performed to estimate double-stranded (ds)DNA concentration of the samples. Samples were not diluted and Quant-IT PicoGreen reagent was added to the wells. Fluorescence signals were detected on the Infinite 200 PRO multimode plate reader (Tecan Group Ltd., Switzerland) with filter settings of 485 nm and 535 nm.

### Enzyme-Linked Immunosorbent Assay (ELISA) Detecting MPO-DNA Complexes

NETs-associated MPO-DNA complexes were quantified using an adapted sandwich ELISA. 96-well flat-bottom plates were coated with goat anti-human/mouse MPO antibody (3,125.10^3^ mg/ml, R&D Systems, AF3667) in PBS overnight at 4°C. The day after, plates were blocked with PBS-Bovine Serum Albumin 1% (BSA, Sigma, A7906) and samples were added. In order to increase the accessibility of MPO-DNA complexes by antibodies and, hence, their detection, we added low amounts of DNAse I (RNase free, 125 UI, Sigma, 11284932001) to the samples for 15 minutes, as previously described (63). To determine the optimal amount of DNAse to use, we added increasing concentration of DNAse (62.5 UI, 125 UI, 250 UI, 500 UI and 1000 UI per well) in supernatants from PMA-stimulated neutrophils and compared the optical density (OD) obtained with the distinct DNAse concentrations. OD reached a plateau between 125 and 250 UI before drastically decreasing with higher DNAse concentrations, likely due to NETs degradation (data not shown). We chose the concentration of 125 UI to maximize the effectiveness of our measurements and to avoid any NETs degradation in our samples. After 15 min, we stopped the enzymatic reaction by adding 1 µl PBS-EDTA (0.05 M) and plates were incubated for 90 min at room temperature. Mouse anti-DNA detection antibodies (1.10^-2^ µg/ml, clone BV16-13, Sigma-Aldrich, MAB030) were added and incubated for 1 hour. Biotinylated polyclonal rat anti-mouse IgG2a (1.10^-2^ µg/ml, BD Biosciences, 553388) were then added for 90 min. Plates were then washed and strepavidin-conjugated horseradish peroxidase (HRP) (1:500 dilution, ThermoFisher, 18-4100-94) was added for 30 min. After washing, the plate was incubated in the dark with 3,3’,5,5’ Tétraméthylbenzidine (TMB, Lifetechnologies, SB02) substrate, and the enzymatic reaction was stopped with H_2_SO_4_ 1 M. Absorbance was measured at 450 nm with a plate reader Multiskan FC (ThermoScientific, 51119000). Between each step until the addition of TMB, 3 to 5 rinses were performed with a wash solution of PBS-Tween-20 5% (ThermoFisher, 233360010).

### Immunofluorescence Stainings and Analysis

Immunofluorescence stainings have been performed on BALF cytospsins and culture slides of blood neutrophils. The cytospin preparations were made by adding 150 µl of BALF to a cytospin funnel, which was then centrifuged at 1000 rpm (112 x *g*) for 4 minutes (rotor JC 301 Cellspin I 1206-14, Tharmac). Culture slides were recovered after supernatants collection and washing wells with PBS. Cytospins or culture slides were fixed in 4% paraformaldehyde and permeabilized with 0.5% Triton X-100, in PBS. Samples were incubated with a blocking buffer (PBS-BSA 2% and FBS 2%; Sigma-Aldrich) for 1 hour at room temperature and stained with rabbit anti-human Cit-H3 (Abcam, Ab5103; 1:100 dilution in blocking buffer) and with goat anti-human MPO (R&D Systems, AF3667; 1:40 dilution in blocking buffer) for 1 hour at room temperature. After washing samples with PBS, secondary donkey anti-rabbit Alexa Fluor 568 (ThermoFisher, A10042, 1:200 dilution in blocking buffer) and donkey anti-goat Alexa Fluor 488 (ThermoFisher, 11055, 1:200 dilution in blocking buffer) were added in blocking buffer containing 4’,6-diamidino-2-phenylindole (DAPI, ThermoFisher, D3571, 1:1000 dilution) and incubated for 2 hours in the dark at room temperature. Finally, samples were mounted with 10 µl of ProLong Antifade reagent (Thermo Fisher, P36961) on glass slides and stored at room temperature in the dark overnight. All samples were analyzed by fluorescence microscopy using standard filter sets. Controls were stained with secondary antibodies after incubation with sera from host species (i.e., rabbit and goat sera, 2% in PBS) without primary antibodies, and nonspecific fluorescent staining was not detected under these conditions. Images were acquired on a Zeiss LSM 880 Airyscan Elyra S.1. confocal microscope (Zeiss) and processed using ImageJ software.

**Figure 1:**
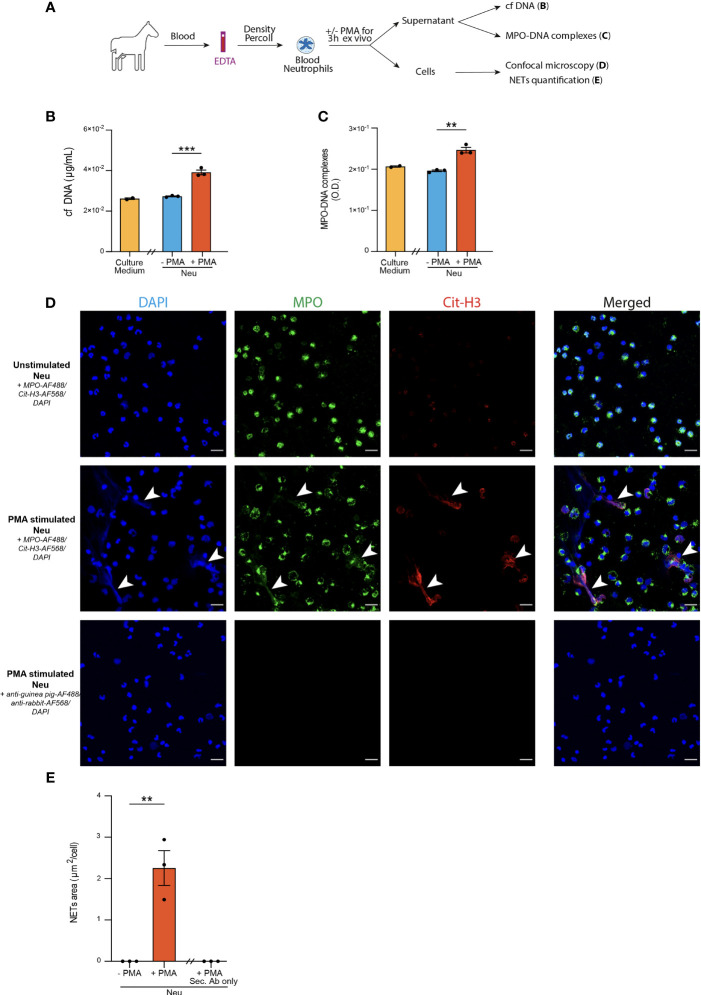
Optimization of three distinct methods to detect NETs release from blood neutrophils in horses. **(A)** Experimental Outline. Horse blood neutrophils were isolated by density gradient and cultured for 3 hours without (negative controls, Neu -PMA) or with (positive controls, Neu +PMA) PMA. Cell culture supernatants were recovered to measure cf DNA and MPO-DNA complexes, and cells were analyzed by confocal microscopy. **(B)** Cf DNA content in culture supernatants evaluated by PicoGreen assay. **(C)** MPO-DNA complexes measured in culture supernatants by ELISA. **(D)** Representative confocal microscopy pictures (objective 20x) of blood neutrophils in the indicated conditions. NETs are defined as extracellular structures double positive for MPO and Cit-H3 and are indicated by white arrows. **(E)** NETs quantification in culture slides expressed as extracellular areas positive for MPO and Cit-H3. **(B, C, E)** Data show mean +/- s.e.m, and individual values from 3 independent experiments. Each symbol represents the mean of one independent experiment. *P* values were estimated with a two-tailed unpaired Student *t*-test. ^**^, *P* < 0.01; ^***^, *P* < 0.001. Neu, neutrophils. Scale bar = 20 µm.

To quantify NETs in BALF, as described previously (28, 31), we acquired six fields per slides (magnification 20x, dry objective). Then, we performed an analysis of structures that were double positive for Cit-H3 and MPO in the Fiji-ImageJ software using one script. Briefly, we defined threshold manually for green and red channels. Then the script replaced each pixel in an image with a black pixel if the image intensity was greater than the defined threshold. The script then produced an image “red inter green”, measured the area of the intersection (i.e., pixels with colocalization) as well as the total number of cells in the picture. Finally, we divided the area of colocalization by the number of cells in the picture to normalize the measurements and we exported the result as a.csv (Comma-separated values) file. This script can be found in the following link: https://github.com/Alexh3g0/NETs-quantification/tree/main. Using this script, we obtained NETs area for each field analyzed. We then corrected the NETs area by the number of cells per field (**Figures 1**, **3**, **5**) or by the number of neutrophils (identified as MPO^+^ cells) (**Figure 3**) and calculated the mean of NETs area/cell or NETs area/neutrophil for each individual.

### Statistical Analyses

**Figure 2:**
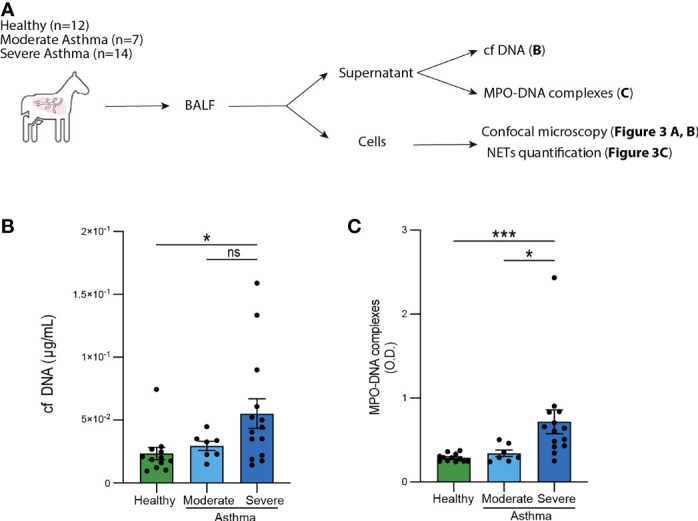
Elevated levels of cf DNA and MPO-DNA complexes in BALF from severe asthmatic horses. **(A)** Experimental Outline. BAL were performed, cytospins were processed and analyzed by confocal microscopy, while supernatants were recovered to measure cf DNA and MPO-DNA complexes. **(B)** BALF cf DNA content measured by PicoGreen assay. **(C)** BALF MPO-DNA complexes measured by ELISA. **(B, C)** Data show mean +/- s.e.m., as well as individual horses (healthy n=12; moderate asthma n=7; severe asthma n=14). *P* values were estimated with a one-way ANOVA with Kruskal-Wallis’ *post hoc* test. ^*^ *, P* < 0.05; ^***^, *P* < 0.001; ns, not significant.

**Figure 3:**
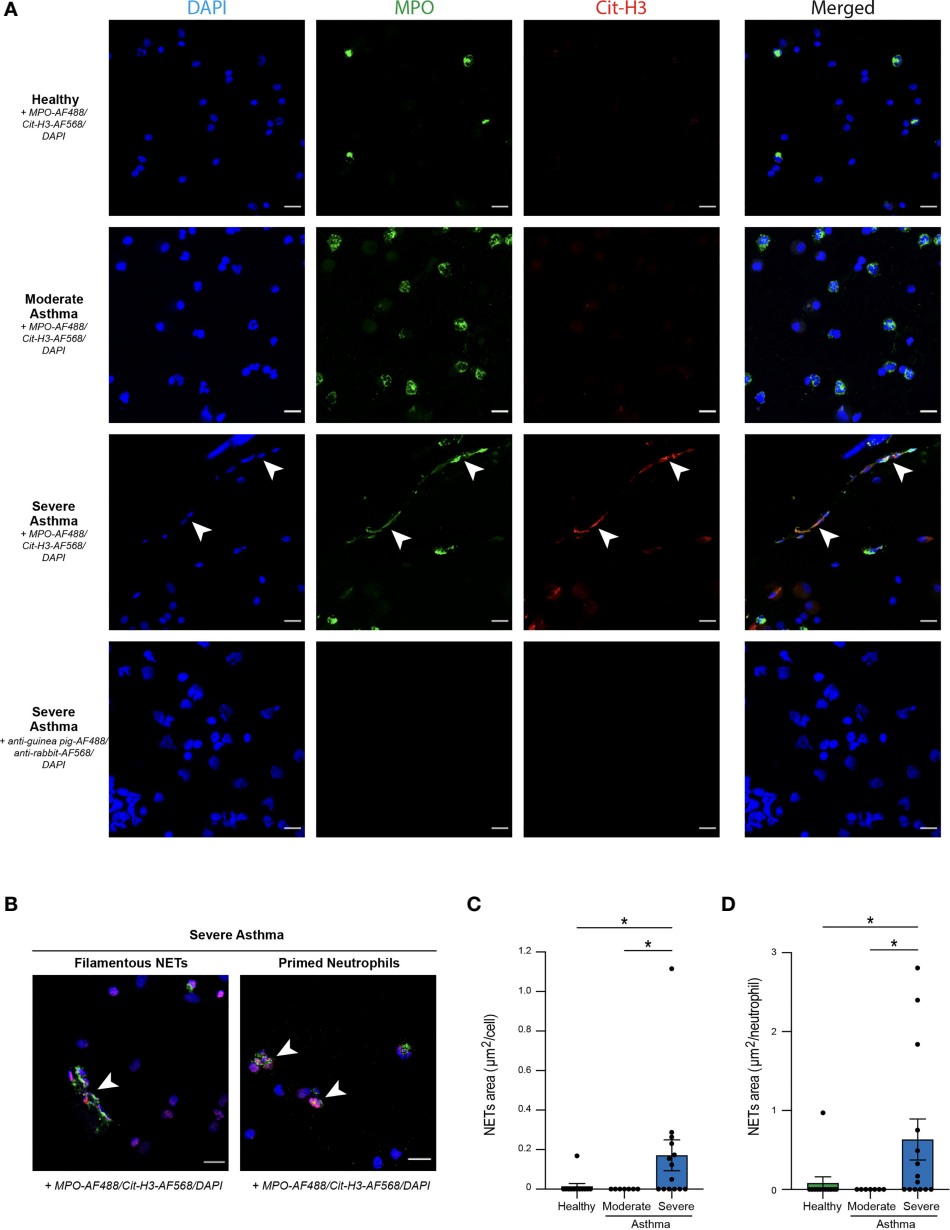
Propensity of BALF neutrophils to release NETs in severe asthmatic horses. **(A)** Representative confocal microscopy pictures of BALF cytospins stained with anti-MPO and anti-Cit-H3 antibodies, and DAPI. NETs are extracellular structures staining double positive for MPO and Cit-H3 and are indicated by white arrows. **(B)** Representative pictures of filamentous NETs and “primed” neutrophils present in BALF of severe asthmatic horses. **(C)** NETs quantification in BALF cytopsins expressed as NETs area per cell. **(D)** NETs quantification in BALF cytospins expressed as NETs area per neutrophil. Data show mean +/- s.e.m., as well as individual horses (healthy n=12; moderate asthma n=7; severe asthma n=14). *P* values were estimated with a one-way ANOVA with Kruskal-Wallis’ *post hoc* test. ^*^ *, P* < 0.05. **(A, B)** Scale bar = 20 µm; objective: 20x.

The results were analyzed with GraphPad Prism^®^ 9.3 (GraphPad Software Inc.). Data in **Figures 1**, **2**, **3**, **5** are presented as mean ± s.e.m. and individual values and were checked for normality distribution using Shapiro-Wilk normality test. As indicated in the respective figure legends, for data that were normally distributed, we used two-tailed unpaired Student *t*-test or a one-way ANOVA with Tukey’s *post hoc* tests for multiple comparisons. Data that were not distributed normally were analyzed with a Mann-Whitney test or Kruskal-Wallis test for pairwise comparisons. Spearman correlation tests were used to evaluate the correlation between NETs read-outs, BALF neutrophil percentages and WCS. *P* values <0.05 were considered statistically significant.

## Results

### Clinical Presentation and Classification of Asthmatic Horses

Horses with a history of cough, nasal discharge or poor performance were considered to be enrolled in our study and examined by a veterinarian. Based on the inclusion criteria, 21 horses with equine asthma were included in our study. According to criteria elaborated by the Equine Asthma Group, a diagnosis of severe versus moderate equine asthma was made based on history, physical examination, tracheal mucus score evaluated by endoscopy and BALF cytology. History, clinical signs and BALF cytology of healthy controls and horses with equine asthma are reported in **Table 1**, **2**, respectively. Out of the 21 asthmatic horses, 14 were classified as suffering from severe equine asthma (3 mares and 11 geldings, aged 12.7 ± 5 [mean ± SD]) and 7 from moderate equine asthma (1 mare, 4 geldings, 2 stallions, aged 9 ± 2.3 [mean ± SD]). Twelve horses (7 mares and 5 geldings, aged 10.1 ± 7 [mean ± SD]) were included in the control group. Severe asthmatic horses had a history of chronic cough, poor performance and/or nasal discharge. Except for one subject they all presented abnormalities during physical examination like cough, nasal discharge, lung crackles and wheezes. Among the 14 severe asthmatic horses, 8 subjects presented labored breathing at rest. All of the severe asthmatic horses, apart from one subject, presented more than 20% of neutrophils in BALF cytology and a mucus score of at least 3/5. Moderate asthmatic horses had a history of poor performance or occasional cough. They presented a normal physical examination or few clinical signs such as a positive cough reflex, or a nasal discharge for two subjects. They all present a normal breathing pattern. Neutrophil count in the BALF were under 20% of total cells and they all presented a mucus score of 2/5, except one subject in which mucus score was 3/5.

### Optimization of NETs Detection Methods on *Ex Vivo* PMA-Stimulated Equine Blood Neutrophils

First, we sought to optimize three NETs detection methods on blood neutrophils isolated from healthy horses and stimulated ex vivo with PMA, a phorbol esther known to induce NETs formation (64). After stimulation, supernatants were recovered to assess levels of cf DNA and MPO-DNA complexes, whereas neutrophils were stained to detect MPO^+^Cit-H3^+^ extracellular NETs by confocal microscopy (**Figure 1A**).

As compared to the supernatants of unstimulated neutrophils, supernatants of PMA-stimulated neutrophils contained higher levels of cf DNA (**Figure 1B**). We did not detect any differences in the amounts of cf DNA in the supernatants of unstimulated neutrophils and the culture medium alone (**Figure 1B**), suggesting that cf DNA detected in supernatants of stimulated neutrophils was derived from NETs and not from neutrophil cytolysis.

Next, we sought to perform a quantification of MPO-DNA complexes present in the supernatants of neutrophil cultures by an in-house adapted sandwich ELISA. Interestingly, we found significantly higher amounts of MPO-DNA complexes in culture supernatants of blood neutrophils stimulated with PMA as compared to unstimulated neutrophils, which exhibited similar OD levels than the background OD levels measured for the medium alone (**Figure 1C**). Thus, using these two methods, we were able to observe significant differences between positive and negative controls, even though some unspecific background was found in medium alone or in unstimulated neutrophils for the MPO-DNA complexes (**Figures 1B, C**).

Finally, we performed immunofluorescence stainings on neutrophil-seeded slides to detect NETs as extracellular structures with a colocalization between MPO and Cit-H3 can be found. We also stained slides of PMA-stimulated neutrophils with secondary antibodies only to confirm antibody staining specificity (**Figure 1D**). Interestingly, we were only able to detect NETs structures in PMA-stimulated neutrophils, but not in unstimulated neutrophils (**Figure 1D**). Furthermore, to perform an objective quantification of NETs areas, we used an algorithm adapted from our previous work in mice and humans (17, 28, 31) to measure areas of MPO^+^Cit-H3^+^ extracellular NETs. Of note, we observed a significantly higher surface of NETs per cell in slides seeded with PMA-stimulated neutrophils as compared to those seeded with unstimulated neutrophils, or those seeded with PMA-stimulated neutrophils but stained with the secondary antibodies only (**Figure 1E**).

Altogether, our data confirm that ELISA quantifying MPO-DNA complexes and confocal microscopy analysis of immunofluorescently-labeled cells represent useful and validated methods to quantify NETs in biological fluids and in seeded neutrophils in the horse species, respectively.

### BALF of Severe Asthmatic Horses Contain Elevated Levels of cf DNA and MPO-DNA Complexes

To investigate the presence of NETs in equine asthma, we performed a BAL on 12 healthy horses, 14 severe asthmatic horses and 7 moderate asthmatic horses. BALF supernatants were used to perform cf DNA and MPO-DNA complexes measurements, while BALF cells were used for NETs detection by confocal microscopy (**Figure 2A**). First, even though we did not find any significant difference in BALF cf DNA concentrations between healthy and moderate asthmatic horses we, found that BALF cf DNA concentrations were significantly higher in severe asthmatic horses as compared to healthy controls (**Figure 2B**). We did not observe any significant difference in cf DNA contents between moderate and severe asthmatic horses, even though severe asthmatic horses displayed a tendency of higher DNA concentrations as compared to moderate asthmatic horses (**Figure 2B**). Second, we measured MPO-DNA complexes by ELISA and found significantly higher levels of MPO-DNA in BALF supernatants of horses suffering from severe asthma as compared to those suffering from moderate asthma and to healthy controls (**Figure 2C**). Noteworthy, we were not able to detect any significant differences between BALF supernatants of healthy and moderate asthmatic horses (**Figure 2C**). Altogether, our data show that amounts of cf DNA and MPO-DNA complexes were higher in BALF supernatant of severe asthmatic horses as compared to healthy horses, while only MPO-DNA levels, not cf DNA levels, were significantly higher in severe asthmatic horses as compared to moderate asthmatic horses.

### Quantification of NETs Structures Reveals a High Propensity of BALF Neutrophils From Severe Asthmatic Horses to Release NETs, Unlike Those From Healthy and Moderate Asthmatic Horses

To further investigate NETs in our samples, we sought to proceed to a direct visualization of extracellular MPO^+^Cit-H3^+^ NETs structures on cytospins from BALF cells. No staining was observed in samples incubated with secondary antibodies only, confirming staining specificity (**Figure 3A**). Interestingly, we were only able to identify NETs in BALF samples isolated from severe asthmatic horses (**Figure 3A**). While NETs were present in their filamentous form in most of the severe asthmatic samples, we also detected NET-prone “primed” neutrophils, characterized by nuclear colocalization of Cit-H3 and DAPI (**Figure 3B**). To objectively quantify NETs release, we acquired six images per sample and determined, using a script developed in our laboratory, the total NETs area per field, reported to the number of cells per sample. We thus obtained a mean of NETs area per cell and found a significantly higher NETs area in BALF of severe asthmatic horses as compared to healthy controls and moderate asthmatic subjects (**Figure 3C**). Finally, we assessed whether higher NETs areas in severe asthmatic horses were due to a higher neutrophil count on those slides, or whether NETs release by BALF cells was a prominent feature of severe equine asthma *per se*. We thus expressed NETs areas per neutrophil present on each analyzed field, neutrophils being identified as cells double positive for DAPI and intracellular MPO. Applying such correction did not modify the results and severe asthmatic horses still exhibited significantly higher NETs area/neutrophil as compared healthy controls or moderate asthmatic horses (**Figure 3D**). Our data support that BALF neutrophils from severe asthmatic horses are particularly prone to release NETs as compared to those from moderate asthmatic horses or healthy controls.

### NETs Levels Correlate With Disease Severity and Neutrophil Percentage in BALF

**Figure 4:**
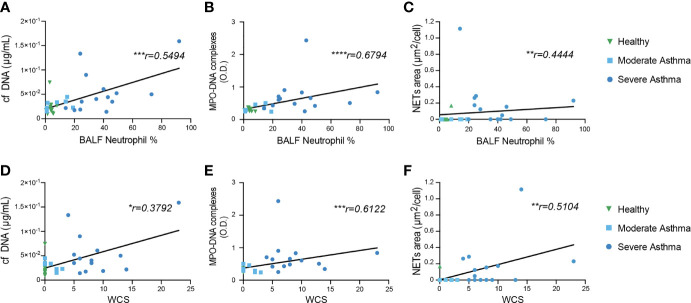
BALF NETs correlate with BALF neutrophil percentage and disease severity score. **(A–C)** Correlation of **(A)** cf DNA, **(B)** MPO-DNA complexes and **(C)** NETs area/cell with BALF neutrophil percentage (%). **(D–F)** Correlation of **(D)** cf DNA, **(E)** MPO-DNA complexes and **(F)** NETs area/cell with disease severity (Weighted clinical score [WCS]). The correlation analysis used was non-parametric (Spearman’s correlation) performed on healthy control and asthmatic horses pooled into a single group. ^*^ *, P* < 0.05; ^**^, *P* < 0.01; ^***^, *P* < 0.001.

Accumulating evidence supports the implication of NETs in the pathophysiology of severe neutrophilic asthma or allergic asthma in human (54, 58, 65–67), and NETs correlate with asthma severity (66, 68) and exacerbations (50) in human. In line with a previous report (43), we observed that neutrophil percentage in BALF positively correlated with disease severity evaluated by WCS (60) (**Supplementary Figure 1**). We sought to investigate the association between NETs levels and the percentage of BALF neutrophils or the disease severity based on WCS. First, we observed that NETs levels, quantified by the 3 methods, positively correlated with the percentage of BALF neutrophils (**Figures 4A–C**). Second, we also found a significant positive correlation between NETs levels and disease severity (**Figures 4D–F**). These observations demonstrate the association of NETs, regardless of the detection method, with the level of neutrophilic inflammation of the airways and the degree of disease severity.

### No Evidence of BALF NETs Release in Moderate Equine Asthma Associated With Neutrophilic Infiltration

**Figure 5:**
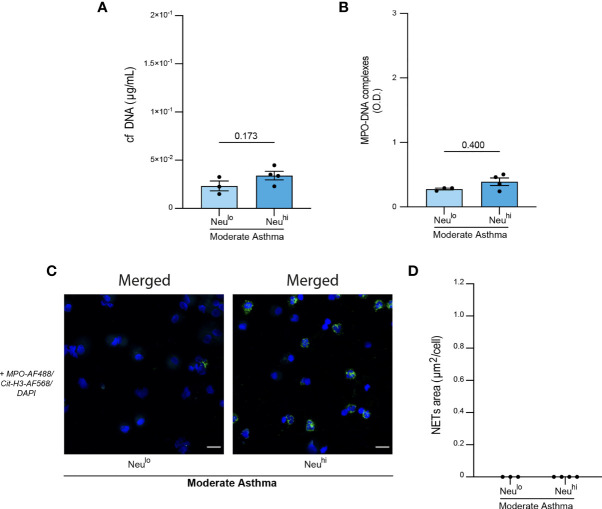
Evidence that BALF neutrophils from moderately asthmatic neutrophilic horses release minimal amounts of NETs. **(A)** BALF cf DNA content measured by PicoGreen assay. **(B)** BALF MPO-DNA complexes measured by ELISA. **(C)** Representative confocal microscopy pictures of BALF cytospins stained with anti-MPO, anti-Cit-H3 antibodies, and DAPI. **(D)** NETs quantification in BALF cytopsins expressed as NETs area per cell. Data show mean +/- s.e.m., as well as individual horses (non-neutrophilic moderate asthma [Neu^lo^] n=3; neutrophilic moderate asthma [Neu^hi^] n=4). *P* values were estimated with a two-tailed unpaired Student *t*-test. **(C)** Scale bars = 20 µm; objective: 20x.

Moderate equine asthma is not always characterized by a mild neutrophilic inflammation of the airways. Indeed, mastocytic or eosinophilic influx have also been reported in the lower airways of moderate asthmatic horses (10, 11, 69, 70). So far, moderate asthmatic horses were classified in one single category. Nevertheless, when considering the percentage of neutrophils in BALF cytology (**Table 2**), moderate asthmatic horses could be sub-classified into two distinct groups: neutrophil^low^ (Neu^lo^, < 5% BALF neutrophils) and neutrophil^high^ (Neu^hi^, > 5% BALF neutrophils). Hence, we then wondered whether NETs release was more prominent or not in moderate Neu^hi^ asthmatic horses as compared to the Neu^lo^ counterpart. We did not observe any difference in cf DNA levels (**Figure 5A**) or amounts of MPO-DNA complexes (**Figure 5B**) between the Neu^lo^ and Neu^hi^ subgroups. Moreover, we did not detect any NETs structures in any of the subgroups, in line with the data shown in **Figures 3C** (**Figures 5C, D**). These analyses, even though performed on a limited number of animals, are consistent with the idea that neutrophils recruited in the airways from moderately asthmatic horses are less prone to release NETs than those from severe asthmatic horses.

### NETs Can Be Used as a Predictive Biomarker for Severe Equine Asthma

**Figure 6:**
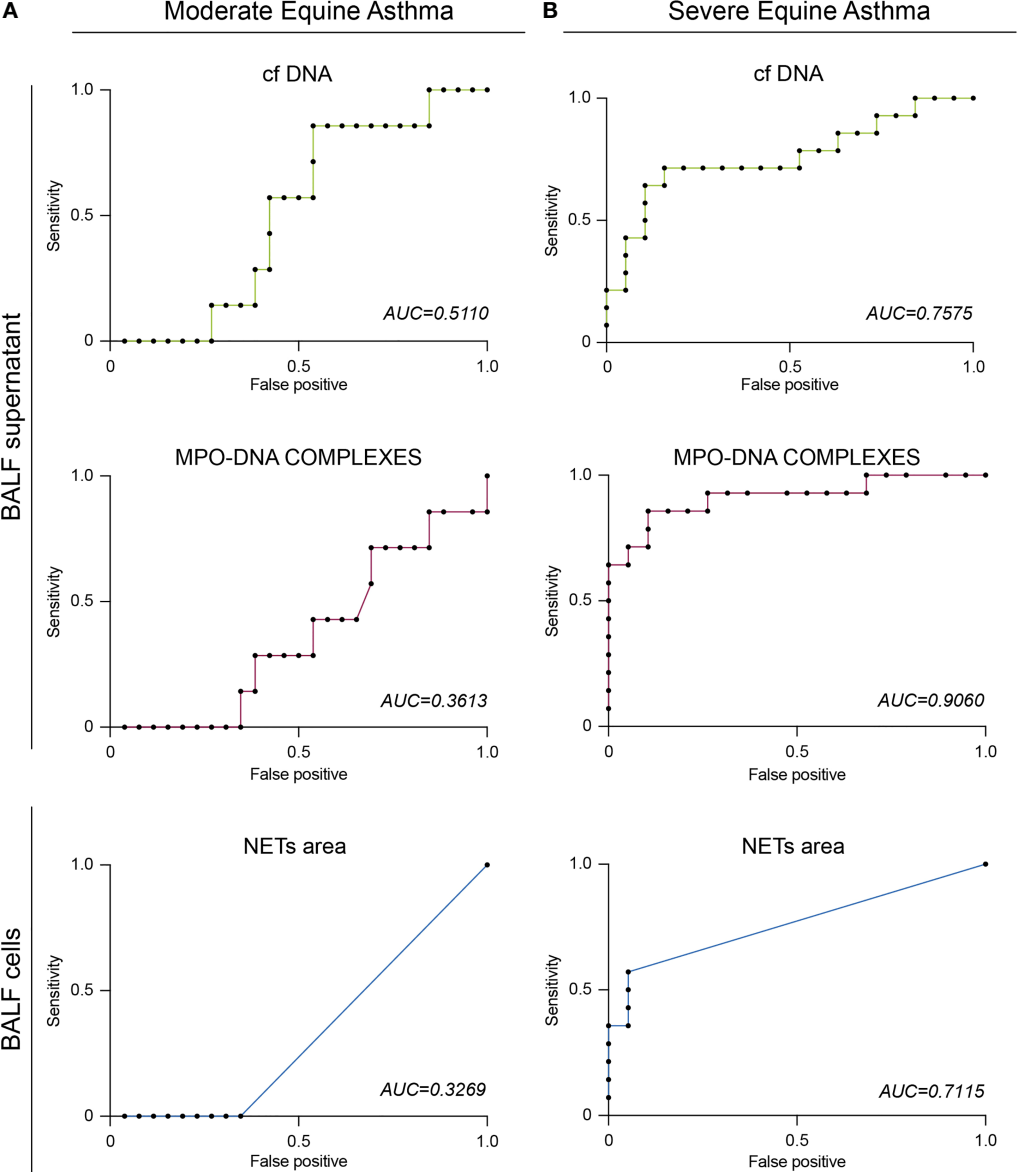
BALF NETs can predict severe asthma in horses. **(A)** ROC curve of BALF cf DNA, MPO-DNA or NETs area for moderate equine asthma. **(B)** ROC curve of BALF cf DNA, MPO-DNA or NETs area for severe equine asthma. AUC, Area Under the Curve.

Finally, we wondered whether the NETs detection methods employed in this study could be useful to predict severe equine asthma versus moderate equine asthma. As expected, using receiver operating characteristic (ROC) curves, we found that BALF neutrophil percentages and the disease score WCS were highly predictive of severe equine asthma (**Supplementary Figure 2**). To determine the predictivity of NETs detection for moderate or severe equine asthma, we analyzed results obtained for each NETs detection methods on BALF samples of severe or moderate horses using ROC curves. For moderate equine asthma, cf DNA showed the best area under the curve (AUC) of 0.5110, whereas AUC curve for MPO-DNA complexes and NETs areas were 0.3613 and 0.3269, respectively (**Figure 6A**). These results support that, more the technique used is specific for NETs, less it is predictive for moderate equine asthma. These data support that NETs, regardless of the detection method used, were a poor predictive biomarker for moderate equine asthma. Conversely, for severe equine asthma, NETs evaluated by MPO-DNA ELISA showed the best AUC of 0.9060, followed by cf DNA (0.7575) and NETs areas (0.7115) (**Figure 6B**). All the three methods of NETs detection used in this study hence provided very good predictivity for severe equine asthma. These data support the idea that NETs, regardless of their method of detection, is a good predictive marker for severe equine asthma and could be used to distinguish severe from moderate equine asthma.

## Discussion

Equine asthma is a highly prevalent chronic pulmonary disorder in veterinary medicine. In horses, two main phenotypes of the disease are described based on history, physical examination, mucus score and BALF cytology, i.e. severe and moderate equine asthma. Yet, the current lack of mechanistic understanding of disease physiopathology that drives disease phenotypes hampers the clinical management and the development of novel, more targeted therapies.

In this study, we enrolled asthmatic horses presented at our faculty as referred cases by practitioners. Such enrollment process presents some limitations. First, as healthy horses are classically not referred to our clinic, we had to recruit healthy horses from the teaching horse herd of the University of Liège, from horses owned by the staff members of the University of Liège and from client-owned horses referred for routine fitness check-ups. In these conditions, it remains sometimes challenging to distinguish a horse with equine asthma in remission from a healthy horse. Nevertheless, asthmatic horses in remission are more likely to respond to at least one of the following criteria: 1) previous history of respiratory symptoms, 2) previous treatment with corticosteroids and/or bronchodilators, and 3) be in a situation of “environmental antigen eviction” which could have masked asthma symptoms. We were careful to exclude horses belonging to any of these categories, as we selected control horses with a well-known history of no respiratory symptom on a long-term basis. Of note, even though one of the healthy horses displayed 8% neutrophils in its BALF cytology, it never experienced any respiratory symptoms. Furthermore, the horses from the teaching herd had previously been submitted to BAL and their cytology was normal at each sampling time. Finally, we had the respiratory cytology available for client-owned horses referred for routine check-up. The ideal test which could have been performed to exclude asthmatic horses in remission would have been a hay challenge test, which is arguably hardly feasible. Of note, all the healthy horses were stabled indoor on straw and fed hay, and none of them was showing any clinical or endoscopic abnormalities at the time of sampling. Second, we had fewer horses with moderate asthma, as these horses were probably more easily managed by the treating veterinarians and less frequently referred to our practice than chronic, severe cases of equine asthma. Nevertheless, the data related to the moderate asthmatic horses were consistent and exhibited a low variability. Third, the personnel in charge of the asthmatic horses enrolled in the study firmly disclosed that the horses had not received any treatment in the 10 days preceding the consultation, but was not able to make that claim beyond 10 days. While we acknowledge that a steroid treatment administered 10 days before presentation might affect the results presented in this study, it was unfortunately not possible to better control for this parameter.

In our cohort, we identified BALF NETs as a hallmark feature of severe equine asthma, as opposed to moderate equine asthma in which NETs were barely detected. We described three distinct methods applicable to BALF, namely detection of cf DNA and quantification of MPO-DNA complexes in supernatants, and quantification of NETs area on cell-seeded slides by confocal microscopy. First, while cf DNA quantification using Quant-It PicoGreen was the quickest method, it is the least specific one since cf DNA is not uniquely found in NETs but could also be triggered by cell damage or death. Second, we optimized the measurement of MPO-DNA complexes by ELISA, which can be used as a high-throughput tool for NETs quantification. The relevance of MPO-DNA complexes as a surrogate for NETs has been debated in the literature (71), since MPO is a positively charged secreted protein that could also bind to negatively charged cf DNA released upon cell damage. Nevertheless, a strength of our study is the use of three distinct methods carrying their own limitations but all converging to the same consistent results, strongly indicating that each of them provided a signal that was specific for NETs in BALF of severe asthmatic horses. Third, we also visualized filamentous NETs by confocal microscopy and quantified them using unbiased computational analyses, which in our opinion is close from the gold standard method to quantify NETs release in tissue sections or body fluids (serum, BALF) (32). Even though, in some particular conditions, NETs release can be achieved without the citrullination of histone 3 (72), the visualization of extracellular filamentous MPO^+^Cit-H3^+^ structures is highly indicative of NETs that rely on histone citrullination for their formation. We designed a script quantifying NETs area based only on the co-localization of green (MPO) and red (Cit-H3) signals. Indeed, in previous studies, we were not always able to see extracellular DNA associated to NETs structures in tissue sections as compared to NETs structures generated *ex vivo* (17, 28). Furthermore, when we added blude (DAPI) intersection with red and green channels in our script, we observed decreased signal in BALF cytospins from severe asthmatic horses, despite the obvious vizualisation of filamentous NETs, as well as increased false positive signal in healthy subjects (data not shown), perhaps due to the presence of artefactual red staining located close to nuclei in some of the healthy controls’ slides.

We used anti-human/mouse MPO antibodies rather than anti-horse MPO antibodies (73) for the MPO-DNA ELISA and for immunofluorescence studies. We already successfully set up immunofluorescence and ELISA protocols using the same antibody for mouse tissue and fluids (sera and BALF) (28, 31), and human and equine MPO have previously been shown to share many similarities and exhibit exactly stacking spectra in spectroscopic analysis as well as the same pH dependency for their enzymatic activity (74). Finally, we compared stainings of BALF equine neutrophils obtained with the anti-human/mouse antibodies with those obtained with specific anti-horse MPO antibodies by confocal microscopy and we could not observe differences between the two antibodies (data not shown).

Several methods have been used to evaluate NETs in equine asthma. Indeed, in 2014, Côté et al. identified NETs in BALF of asthmatic horses by detecting a high molecular weight nucleic acid band in electrophoresis gel, by assessing the levels of cit-H3 protein by western blot analysis and by confocal microscopy (33). Furthermore, in 2011, Vargas et al. also investigated NETs release in BALF cells of severe asthmatic horses by confocal microscopy and quantified NETs by attributing a visual NETs score (0: No NETs, 1: Rare NETs, 2: Moderate NETs, 3: Widely distributed NETs) on classical microscopy (34). In 2017, the same group also developed a NETs quantification method based on NETs score from confocal microscopy images of NETs generated *in vitro* (35). To our knowledge, our study describes for the first time two NETs quantification methods by measuring MPO-DNA complexes by ELISA on BALF supernatants and by evaluating NETs area based on colocalization of MPO and Cit-H3 stainings in randomly acquired confocal microcopy images of BALF cells. These two techniques show high specificity and represent valuable assays to evaluate NETs in biological fluids or in tissue sections, respectively.

Our primary material of study was the BALF. As BAL are invasive and could be challenging to perform in horses exhibiting severe symptoms, the identification of blood biomarkers could be very helpful. While we did not assess NETs release in blood samples of the horses enrolled in our study, measurement of cf DNA and MPO-DNA complexes could both be applicable to serum or plasma samples. Of note, alterations of blood neutrophils have been described by Herteman and colleagues in equine asthma (35), which can be related with NETs release. Indeed, they identified a higher proportion of low-density neutrophils (LDNs) in the blood of severely asthmatic horses as compared to healthy controls and showed that such LDNs displayed a higher NETs release potential when compared to normal density neutrophils of the same subjects (35), consistent with the hypothesis that NETs could also be released systemically in severe asthma.

We provided evidence that NETs were only present in BALF of severe asthmatic horses but not in BALF of moderate asthmatic horses, emphasizing the interest of considering NETs as a biomarker to predict severe equine asthma. Other biomarkers have already been proposed, such as acute phase protein or inflammatory cytokines in the serum. Haptoglobin, secretoglobin and surfactant-D protein have already been identified in the blood of severe (33) and moderate (75, 76) asthma, but their relevance to distinguish between the two forms has not been investigated. Furthermore, the use of acute phase protein as a marker of equine asthma has been controversial as some investigators could not observe differences between asthmatic horses and horses with non-respiratory exercise intolerance (77). Indeed, a systemic increase of acute phase protein is rather a general hallmark of inflammation that is independent of the causative agent. Circulating immune complexes have also been proposed as a reliable biomarker to diagnose equine asthma, but levels of circulating immune complexes in severe versus moderate asthmatic horses have never been compared (78). Tissue inhibitor of metalloproteinases (TIMPs) and matrix metalloproteinases (MMPs) could be used for the diagnosis of equine asthma but only MMP-2 and MMP-9 were differentially expressed in severe equine asthma versus moderate equine asthma, MMP-2 levels being higher in moderate equine asthma whereas MMP-9 levels being higher in severe asthmatic horses (79). Finally, exhaled breath condensate is a promising source of biomarkers in human lung diseases. In horses, studies have investigated its use to diagnose lower airway inflammation and showed promising results for severe equine asthma but data for moderate equine asthma are still missing (80). Of note, evaluation of exhaled breath condensate can be difficult to set up on the field. In conclusion, several biomarkers have already been investigated and demonstrated to be reliable to identify one of the two phenotypes of equine asthma as compared to healthy subjects but whether they could be used to unequivocally characterize severe and moderate asthmatic horses has never been investigated.

NETs release can be induced by several factors identified to be differentially expressed in lungs of severe asthmatic horses as compared to moderate asthmatic horses or healthy controls. First, IL-1β, highly expressed in severe equine asthma (38), has been demonstrated to induce NETs release in mouse models of gouty arthritis (81) and abdominal aortic aneurysms (82). In these two models, the use of IL-1β inhibitors or IL-1β-deficient mice were strongly associated with a decrease of NETs release and a relief in disease features, supporting an important role for IL-1β in NETs release and disease physiopathology. Moreover, experiments comparing gene expression of primary bronchial epithelial cell cultures (BECCs) from healthy and severe asthmatic horses have shown an increased expression of *CXCL2* in BECCs of severe asthmatic horses upon stimulation with hay dust or lipopolysaccharide (LPS) (83). As CXCL2 has also been shown to induce NETs release and neutrophil activation *in vitro* (84), it could promote NETs release in lungs of severe asthmatic horses. Finally, Côté and colleagues have shown a decreased secretion of secretoglobin 1A1 in BALF of severe asthmatic horses as compared to healthy subjects. In their study, they also demonstrated an inhibitory role of secretoglobin 1A1 on NETs release by equine neutrophils stimulated *ex vivo* (33). Thus, low levels of secretoglobin in severe asthmatic horses could be insufficient to control NETs release in these subjects. Of interest, moderate asthmatic horses, unlike severe asthmatic horses, had a normal blood concentration of secretoglobin which could explain why high neutrophil numbers found in the airways of moderate asthmatic horses were not associated with NETs release in our study. This last point should be interpreted carefully since in 2019, Gy et al. measured secretoglobin levels in the blood and not in the BALF of moderate asthmatic horses as in the study of Côté published in 2014 (33, 76).

NETs have been implicated in the pathophysiology of both severe neutrophilic and allergic asthma in humans (50, 65, 66, 85). Indeed, NETs have been found in the sputum of severe asthmatic patients (68) where they strongly correlated with disease severity and inflammasome activation (66). In allergic human asthma, NETs have been detected in bronchial biopsies of patients (85) and in nasal lavage samples, where they correlated with allergic asthma exacerbations induced by rhinoviruses (50). Interestingly, we also observed a correlation between NETs and disease severity in our study. Thus, NETs, aside being a biomarker for severe equine asthma, likely emphasize immunopathological changes occurring uniquely in severe asthma, and could thus help to better understand disease physiopathology. NETs in the airways of severe asthmatic horses have indeed the ability to contribute to various pathophysiological aspects of severe equine asthma. First, NETs could promote the release of pro-inflammatory cytokines observed in severe equine asthma. Indeed, NETs can promote the secretion of IL-1β by macrophages in a mouse model of asthma (54) and they have also been demonstrated to contribute to IL-1 activation and processing *in vitro* (86). NETs can also promote secretion of MMP-8 by macrophages *in vitro* (87),which is associated with airway obstruction in BALF of human non-controlled severe asthma (88). Indeed, MMP-8 is usually associated to foci of damaged lung tissue, especially close to damaged epithelium or inflamed epithelial cells, and seems to refer to an onset of irreversible tissue injury rather than acute inflammation. NETs release associated to high levels of MMP-8 in the airways of severe asthmatic horses could be in part responsible for laborious breathing at rest observed in severe equine asthma but not in moderate equine asthma (89). Severe equine asthma is also characterized by high mucus scores observed during endoscopic examination. One the one hand, NETs have also been associated to increased mucus secretion in the airways in a mouse model of acute lung injury induced by exposure to LPS (51). On the other hand, high amount of NETs-associated DNA in mucus makes it more viscous, decreased its transit and elimination from the lower airways and favor its accumulation (90, 91), thereby impairing pulmonary ventilation (16). Finally, increased TLR4 expression on bronchial epithelial cells has been observed in a model of severe equine asthma, along with increased BALF expression of TLR4 in severe asthmatic horses as compared moderate asthmatic horses (38, 92). NETs could thus enhance local inflammation by activating TLR4 expressed by the airway epithelium and then promote chronic inflammation (29), tissue lesions and by this way worsen respiratory symptoms as observed in severe quine asthma.

Moderate equine asthma is not always associated with a mild neutrophilic inflammation of the airways, and two sub-groups have been described according to the nature of innate immune cells infiltrating the airways, i.e. neutrophils or mastocytes/eosinophils (10, 59). Recent studies have identified distinct cytokines signatures associated to each sub-group, with “neutrophilic” moderate equine asthmatic horses showing neutrophilic inflammation exhibiting elevated IL-1β (10, 93) and TNF-α (10) and those with mastocytic/eosinophilic inflammation showing elevated IL-4 (10, 59). IFN-γ (10, 93) and IL-5 (59, 69) can be found in both neutrophilic and mastocytic moderate asthma. Clinical features have also been linked to particular sub-types of moderate equine asthma. Cough has been associated to the neutrophilic form of moderate equine asthma (11), whereas exercise intolerance and airway hyperreactivity have been associated to the mastocytic/eosinophilic form of the disease (11). In our study, we assessed whether NETs release was associated with the neutrophilic form of moderate equine asthma. Of note, we did not observe any differences in NETs release between moderate asthmatic horses showing high neutrophilic inflammation (moderate Neu^hi^) and those exhibiting low neutrophilic inflammation (moderate Neu^lo^). Actually, we were not able to detect nor quantify any filamentous NETs on the cytopsins of BALF of moderate Neu^hi^ horses. It is important to note that, due to the limited number of moderate asthmatic horses enrolled in our study, the results should be interpreted with caution. Nevertheless, our data are consistent with the hypothesis that NETs release is rather a hallmark feature of severe equine asthma than a characteristic of neutrophilic inflammation of the lower airways. It is therefore plausible that neutrophils recruited to the airways of moderate asthmatic horses are less prone to release NETs than those recruited in severe equine asthma, likely reflecting distinct disease microenvironment and physiopathology. These potentially interesting results should however be validated in larger cohorts of moderate asthmatic horses.

In conclusion, NETs can be detected in BALF samples of horses using distinct quantifiable methods. NETs are uniquely found in the airways of severe asthmatic horses, but not of moderate asthmatic horses, and correlate with asthma disease severity. Our findings support distinct physiopathological mechanisms involved in both disease phenotypes, with NETs being a potentially important player in driving severe asthma disease and, hence, a promising therapeutic target.

## Data Availability Statement

The original contributions presented in the study are included in the article/**Supplementary Material**. Further inquiries can be directed to the corresponding author.

## Ethics Statement

All the procedures have been approved by the local Ethical Committee (File references DE2453 for BAL and TW procedures and DE1565 for blood collection). Written informed consent was obtained from the owners for the participation of their animals in this study.

## Author Contributions

CR and TM conceived the project. PJ, IT, CR, and TM were involved in experimental design. IT collected BALF from horses. PJ and IT performed most experiments, analyzed and compiled the data. PJ, TM, and CR prepared the figures. AH developed the script and analyzed the confocal microscopy pictures. PM helped with the isolation of blood neutrophils. CR and TM supervised the study and wrote the manuscript. All authors provided feedback on the manuscript.

## Funding

TM is supported by the FRFS-WELBIO (Walloon Excellence in Life Sciences and Biotechnology), by the Acteria Foundation, and by a European Research Council Starting Grant (grant no. ERC-StG-2018 IM-ID 801823). CR is a "Chargé de Recherche" of the F.R.S.-FNRS and is supported by the Acteria Foundation.

## Conflict of Interest

The authors declare that the research was conducted in the absence of any commercial or financial relationships that could be construed as a potential conflict of interest.

## Publisher’s Note

All claims expressed in this article are solely those of the authors and do not necessarily represent those of their affiliated organizations, or those of the publisher, the editors and the reviewers. Any product that may be evaluated in this article, or claim that may be made by its manufacturer, is not guaranteed or endorsed by the publisher.

## Supplementary Material

Supplementary Figure 1 Disease severity correlates with BALF neutrophil percentage. Correlation of disease severity (Weighted clinical score [WCS]) with BALF neutrophil percentage (%). The correlation analysis used was non-parametric (Spearman’s correlation) performed on healthy control and asthmatic horses pooled into a single group. ^***^, *P* < 0.001.

Supplementary Figure 2 Disease severity score and BALF neutrophil percentage are predictive for severe asthma in horses. **(A)** ROC curve of disease severity and BALF neutrophil percentage for moderate equine asthma. **(B)** ROC curve of disease severity and BALF neutrophil percentage for severe equine asthma. AUC, Area Under the Curve.
