# Supplementary figures and images for: Neutrophil Extracellular Traps Are Found in Bronchoalveolar Lavage Fluids of Horses With Severe Asthma and Correlate With Asthma Severity
Source: Front Immunol. 2022 Jul 13;13:921077. doi: 10.3389/fimmu.2022.921077 (PMC9326094; doi:10.3389/fimmu.2022.921077)

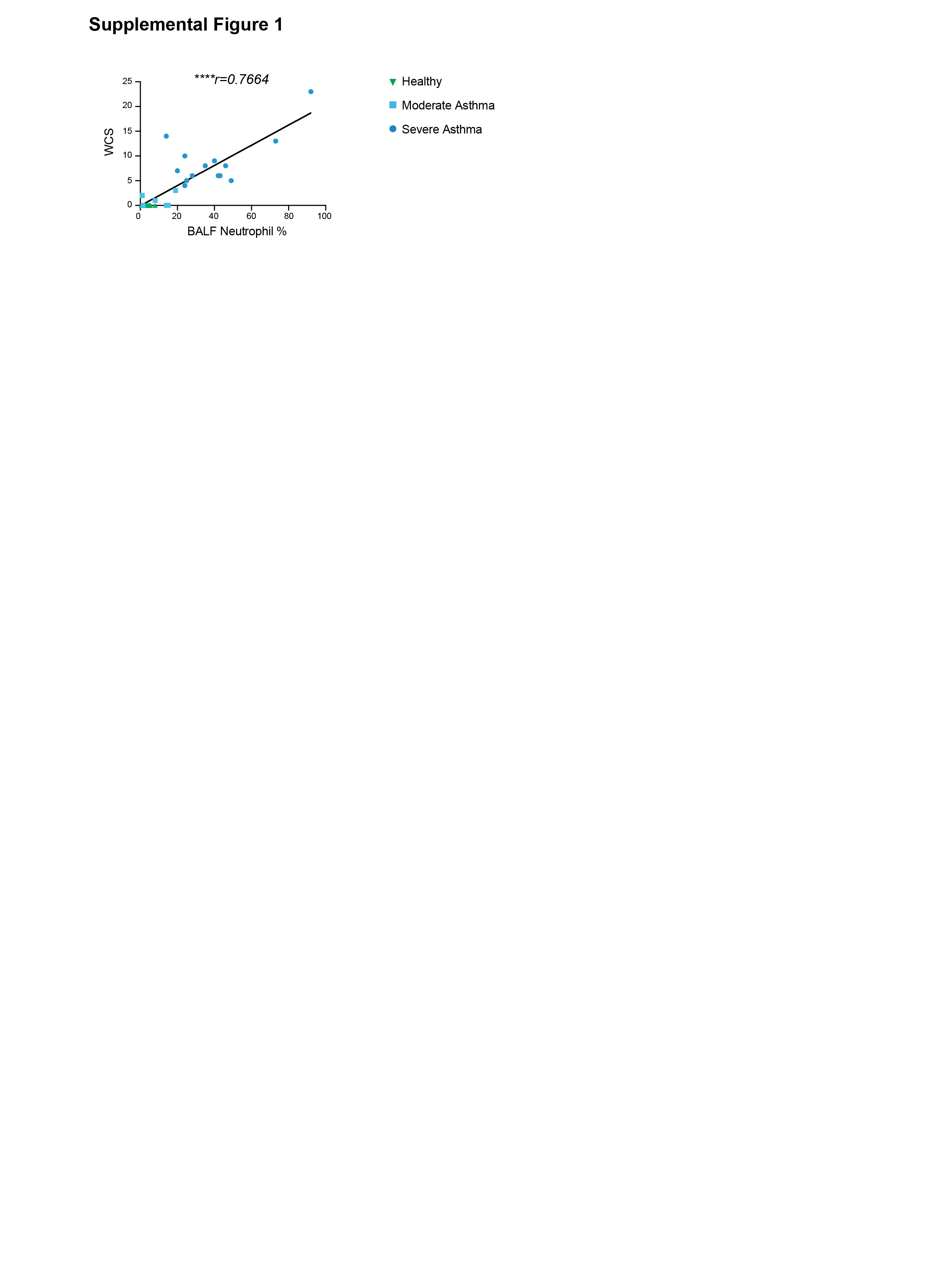

Supplement: Supplementary Figure 1 — Disease severity correlates with BALF neutrophil percentage. Correlation of disease severity (Weighted clinical score [WCS]) with BALF neutrophil percentage (%). The correlation analysis used was non-parametric (Spearman’s correlation) performed on healthy control and asthmatic horses pooled into a single group. ***, P < 0.001. [file Image_1.jpg]

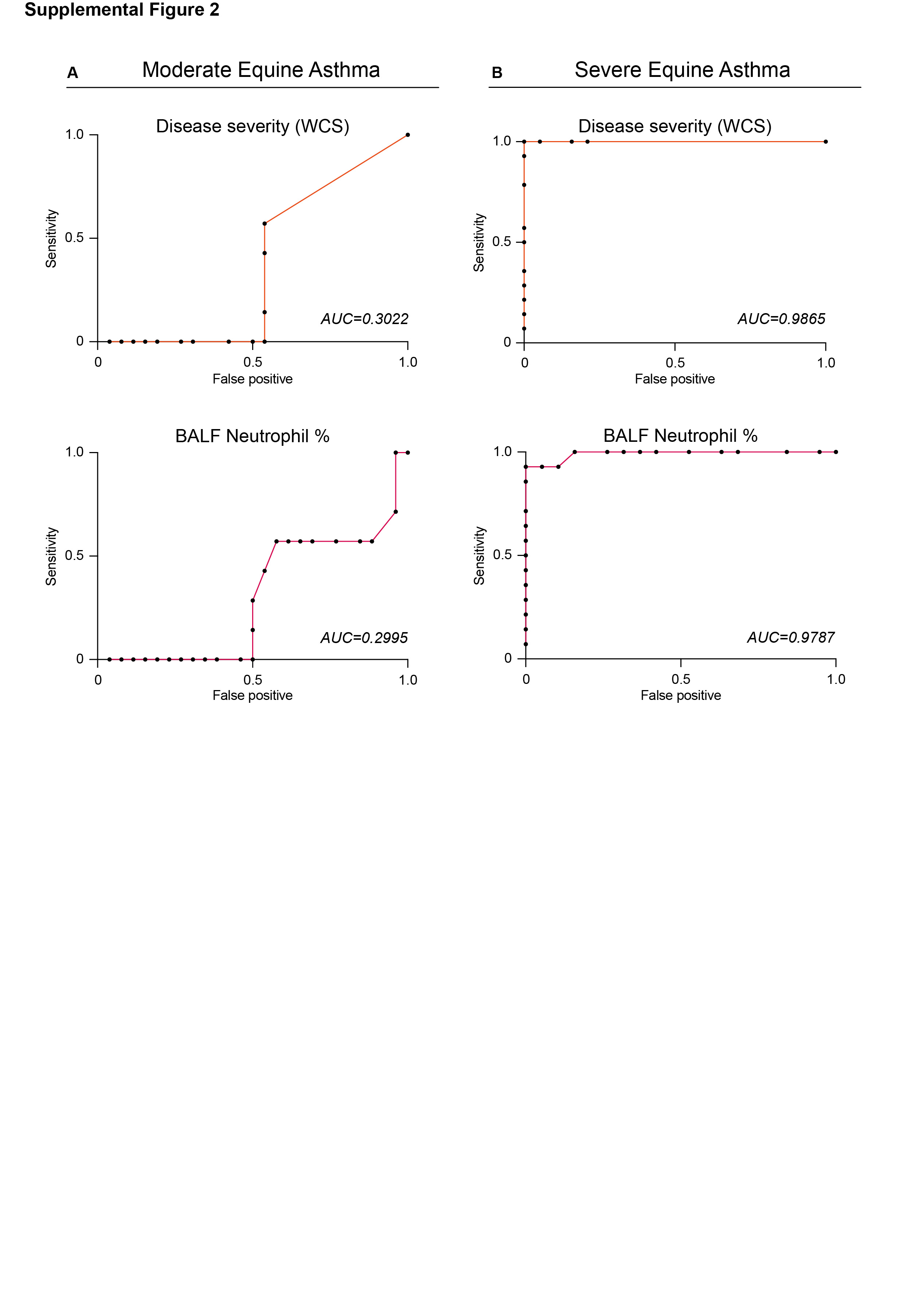

Supplement: Supplementary Figure 2 — Disease severity score and BALF neutrophil percentage are predictive for severe asthma in horses. (A) ROC curve of disease severity and BALF neutrophil percentage for moderate equine asthma. (B) ROC curve of disease severity and BALF neutrophil percentage for severe equine asthma. AUC, Area Under the Curve. [file Image_2.jpg]
